# Effects of single-, dual-, and multi-frequency ultrasound-assisted freezing on the muscle quality and myofibrillar protein structure in large yellow croaker (*Larimichthys crocea*)

**DOI:** 10.1016/j.fochx.2022.100362

**Published:** 2022-06-16

**Authors:** Chuhan Bian, Huijie Yu, Kun Yang, Jun Mei, Jing Xie

**Affiliations:** a College of Food Science and Technology, Shanghai Ocean University, Shanghai 201306, China; b National Experimental Teaching Demonstration Center for Food Science and Engineering Shanghai Ocean University, Shanghai 201306, China; c Shanghai Engineering Research Center of Aquatic Product Processing and Preservation, Shanghai 201306, China; d Shanghai Professional Technology Service Platform on Cold Chain Equipment Performance and Energy Saving Evaluation, Shanghai 201306, China

**Keywords:** Ultrasound-assisted freezing, Multi-frequency ultrasound, Large yellow croaker, Freezing rate, Myofibrillar structure

## Abstract

Ultrasound-assisted freezing (UAF) has been proved to be a new technology to improve the quality of frozen foods. Frequency is an important parameter affecting UAF result. This study was to investigate the effects of single-, dual- and multi-frequency UAF on muscle quality and myofibrillar protein structure in large yellow croaker (*Larimichthys crocea*), providing reference for the application of multi-frequency UAF in frozen foods. Multi-frequency UAF increased the freezing rate and had lower thawing loss, thiobarbituric acid reactive substances (TBARS) value, total volatile basic nitrogen (TVB-N) value, and higher immobilized water content. Multi-frequency UAF had lower carbonyl, higher sulfhydryl content, and more stable myofibrillar protein secondary and tertiary structures. Confocal laser scanning microscopy (CLSM) indicated that the filamentous polymer in muscle fibrin solution with multi-frequency UAF was transformed into more evenly distributed units. In general, multi-frequency UAF significantly improved the freezing rate, reduced lipid oxidation, and maintained the myofibrillar structure.

## Highlights

• MUAF significantly promoted the freezing process of large yellow croakers.
• MUAF enhanced the quality of large yellow croakers.
• MUAF better maintained the stability of fish protein.
• The mechanisms of single-, dual-, and multi-frequency UAF were analyzed.

## 1 Introduction

Large yellow croaker (*Larimichthys crocea*) is an important economic marine fish, which is widely cultured in China (Zhang et al., 2021). Freezing is an important preservation method for large yellow croaker during the long-term storage (Li, Li, & Hu, 2013). The ice crystals formed in the freezing process damaged the fish microstructure (Li et al., 2018, Luan et al., 2018). Large and unevenly distributed ice crystals could cause irreversible mechanical damages to the muscle tissue, which destroyed the myofibrillar protein (MP) stability and further affected the function and structure of MP (Wang et al., 2019). Emerging food processing techniques have been reported to effectively maintain the quality of frozen foods by reducing the freezing time, such as pulsed electric fields, high pressure, static magnetic fields, radiofrequency waves, and power ultrasound (Speranza et al., 2021, Tian et al., 2021).

Ultrasound-assisted freezing (UAF) is a new technology that can effectively improve the quality of frozen foods, with the advantage of being able to effectively reduce freezing time (Ma, Mei, & Xie, 2021). The impact of ultrasound on food freezing processes has been reported to be positive, mainly due to its ability to facilitate the nucleation process and increase heat transfer efficiency. The distribution and size of ice crystals played an important role in maintaining the quality of frozen foods. UAF could help to form smaller ice crystals and create a more regular distribution of ice crystals during the freezing process (Daghooghi-Mobarakeh, Subramanian, & Phelan, 2022). Acoustic cavitation was regarded as the main cause of most sono-chemical reactions in liquid systems (González, 2022). Cavitation bubbles were created when ultrasound propagated in liquid–solid systems. The gradual growth and final collapse of the cavitation bubbles led to acoustic cavitation, which caused mechanical effects (turbulence, shear forces, and pressure), chemical effects (free radicals), and thermal effects (temperature) (Zhang et al., 2020). The rapid and violent explosion of cavitation bubbles during the sonication process generated intense physical forces including microjets, shear force, turbulence, and shock waves (Zhang et al., 2020). Compared to conventional ultrasonic cleaners and single-frequency UAF devices, multi-frequency UAF devices could increase the cavitation yield, due to the increased number of mechanical interference and cavitation nuclei in multi-frequency UAF (Zhang et al., 2020). Multi-frequency UAF prevented directional effects in the reactor by providing strong mode disturbances. In contrast, single-frequency UAF reactors were prone to cause directional changes in the ultrasonic field, making it difficult to disperse the energy uniformly in the reaction medium with limited efficiency (Yang, Luo, Wu, Yang, & Kan, 2020). Therefore, the main purpose of this study was to investigate the effects of UAF on the freezing rate, thawing loss, color, water distribution and migration, total volatile basic nitrogen (TVB-N), thiobarbituric acid reactive substances (TBARS), and MP structure of large yellow croakers under single-, dual-, and multi-frequency UAF treatment. This study indicated the effects of single-, dual- and multi-frequency UAF on muscle quality and myofibrillar protein structure in large yellow croaker, providing reference for the application of multi-frequency UAF in frozen foods.

## 2 Materials and methods

### 2.1 Sample pretreatment

The fresh large yellow croaker (500 ± 20 g weight, 30 ± 5 cm long) was obtained from the seafood market in Luchaogang Town, Pudong New Area, Shanghai. Fresh large yellow croaker with gills and viscera removed were purchased and transported to the laboratory within 30 min with ice. The fresh samples were washed with plenty of deionized water and then put into polyethylene bags for vacuum packaging. The packaged fishes were refrigerated in a 4 °C refrigerator, so that the fishes of each experimental group had the same initial temperature before freezing.

### 2.2 Freezing process

**Fig. 1:**
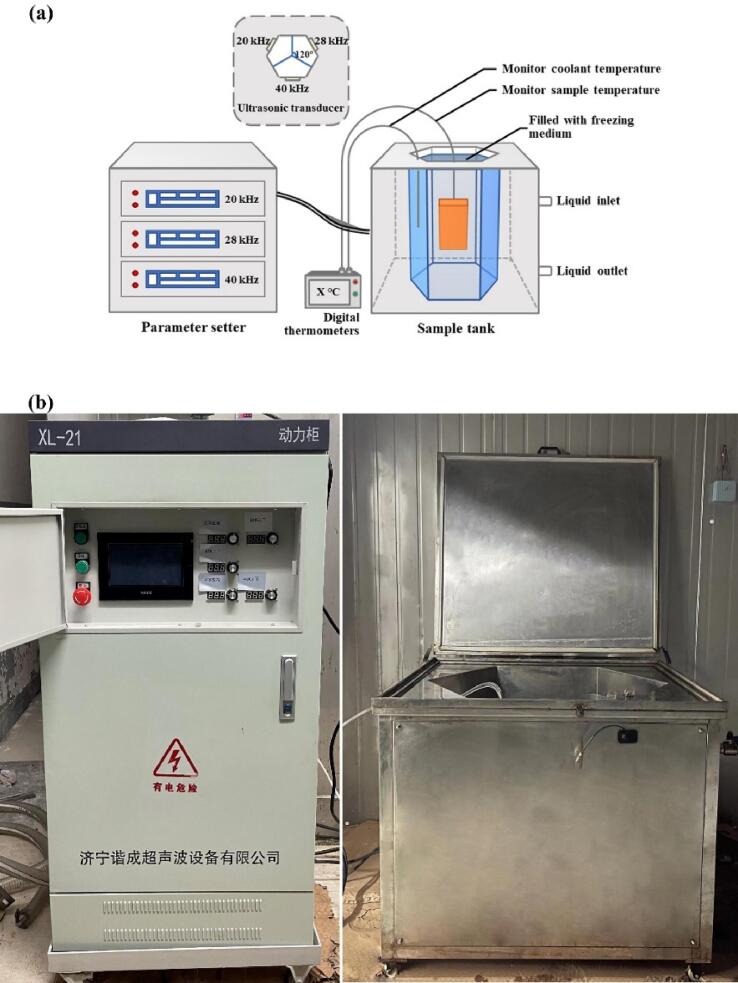
Schematic diagram of multi-frequency ultrasound assisted freezing (UAF) system.

The packaged fish samples were frozen under different treatments until their central temperature reached −18 °C. The multi-frequency UAF instrument was made by Xiecheng Ultrasonic Equipment Co., Ltd. (Jining, Shandong, China) (Fig. 1). There were seven different ultrasound-assisted freezing groups: single-frequency UAF at 20 kHz (SUAF-20), 28 kHz (SUAF-28), and 40 kHz (SUAF-40), respectively; dual-frequency UAF at 20/28 kHz (DUAF-20/28), 20/40 kHz (DUAF-20/40), and 28/40 kHz (DUAF-28/40), respectively; and multi-frequency UAF at 20/28/40 kHz (MUAF-20/28/40). The power of UAF in this research was set to 180 W. 29.3 % calcium chloride brine solution (w/v) was used as the coolant in the UAF system. The coolant temperature was kept at −25.0 ± 0.5 °C using a pump. UAF treatment worked when the central temperature of the samples reached −1 °C (the freezing point of large yellow croaker). It worked for 30 s and took 30 s off for reducing the negative effects of thermal effects. Immersion freezing (IF) was used as the control group, and the fishes were frozen in the coolant at −25.0 ± 0.5 °C. The coolant of IF was also 29.3% calcium chloride brine solution (w/v). The frozen samples were stored in the refrigerator at −20 ± 1 °C for 5 days. The samples were thawed in running water and subsequent experiments were carried out. A networked multipoint temperature collector (Fluke 2640A, Fluke Electronic Instruments Co., Ltd, USA) connected to a *T*-type thermocouple was used to record the central temperature of the large yellow croaker samples in real-time. All indexes were tested with fresh fish group (FS) as control.

### 2.3 Thawing loss determination

Thawing loss was detected according to the method of Zhang, Sun, Chen, Kong, and Diao (2020b). The weight of the samples before freezing is recorded as (W_1_, g). The samples wiped with filter paper were weighed accurately (W_2_, g) after thawing.

$$\mathrm{Thawing}\;loss\;\left(\%\right)=\frac{\left(W_{1}-W_{2}\right)}{W_{1}}\times 100\%$$

### 2.4 Water migration and distribution

The samples were cut into pieces (1.5 × 1.5 × 2.0 cm). The pieces were wrapped using polyethylene films. An LF-NMR analyzer was used to detect the transverse relaxation T2 (Niumag MesoMR23-060H. I, Suzhou, China). Corresponding to the pulse sequence of Carr-Purcell-MeiboomGill, 21 MHz was selected as the frequency of proton resonance. 3000 echoes and 16 scans were included in each measurement. The pseudo color images of proton density-weighted of samples were obtained by performing magnetic resonance imaging (MRI).

### 2.5 Color determination

A colorimeter was used to measure the color of the frozen samples (CR-400, Konica Minolta, Tokyo, Japan). L* (lightness), a* (the red-green degree), and b* (the yellow-blue degree) were detected to analyze the color change of the samples. In addition, the total color difference (ΔE) was calculated using the following formula.

$$\Delta E=\sqrt{{\left(L^{*}-L_{0}\right)}^{2}+{\left(a^{*}-a_{0}\right)}^{2}+{\left(b^{*}-b_{0}\right)}^{2}}$$

### 2.6 TVB-N determination

A Kjeldahl apparatus was used in the TVB-N determination (Kjeltec8400, Foss, Hilleroed, Denmark). The TVB-N value was detected by the microtitration method. The result of the TVB-N was expressed in mg N/100 g.

### 2.7 TBARS determination

TBARS determination was measured referring to the approach of Li et al. (2020). 20 mL of 20% thiobarbituric acid (TBA) solution was homogenized with 5.0 g of sample. The mixture was allowed to rest at room temperature for 60 min. The mixture after homogenization was centrifuged at 11,960×*g* for 15 min. 5 mL of supernatant was mixed with 5 mL of TBA solution (0.02 M). After the above steps, the mixture was heated for 40 min at 100 °C. Finally, the mixture was cooled at room temperature for 30 min. A spectrophotometer was used to measure the mixture absorbance at 532 nm (Evolution 220, Thermo Fisher Scientific, MA, USA). The TBARS value was represented using the content of MDA. The result of the TBARS value was expressed in mg MDA/kg. The absorbance of the sample and the standard was denoted by A_1_ ang A_0_, respectively, and the mass fraction of the sample and the standard denoted by ω_1_ ang ω_0_.

$$\mathrm{TBARS}\;\left(\omega_{1}\right)=\frac{A_{1}}{A_{0}}\times \omega_{0}$$

### 2.8 Extraction of MP

MP solution was obtained according to the method of Tan, Ding, Mei, and Xie (2022). 2 g of sample was homogenized with 20 mL of precooled Tris-buffer A (0.05 M sodium chloride). The mixture was centrifuged for 15 min at 11,960×*g*. After retaining the precipitate, the above steps were repeated and the precipitate was collected. The precipitate was mixed with 20 mL of Tris-buffer B (0.6 M sodium chloride) and stirred evenly at 4 °C. Before being centrifuged at 11,960×*g* for 15 min, the mixture stood for 3 h at 4 °C. The supernatant was retained as an MP solution.

### 2.9 Carbonyl content

The carbonyl content of the MP solution was measured according to the approach of Zhang, Li, Hong, and Luo (2020). Ten millimeters of 2,4-dinitrophenyl hydrazine (DNPH) was mixed with 1 mL MP solution at 25 °C for 1 h. The mixture was precipitated with 1 mL 20% trichloroacetic acid (TCA) solution and centrifuged at 2500×*g* for 15 min at 4 °C. The supernatant was discarded and the precipitate was mixed with 1 mL ethanol: ethyl acetate (1:1, v/v) containing 10 mM HCl. Then the resulting pellet was incubated after dissolving in 6 M guanidine hydrochloride at 37 °C for 16 min. The absorbance was then measured at 370 nm and the free carbonyl compounds content was expressed as µmol/g protein.

### 2.1 Total sulfhydryl content

The total sulfhydryl content of the MP solution was determined according to approach of Li, Mei, and Xie (2021). One gram of large yellow croaker flesh was homogenized with 10 mL 8 M urea and 0.6 M NaCl solutions, and then the mixture was centrifuged at 2500×*g* for 10 min at 4 °C. Supernatant in an amount of 0.5 mL was mixed with 4.5 mL buffer C (pH 8.0, 0.2 M Tris-HCl, 8 M urea, 3 mM EDTA, 1% SDS), and 0.625 mL buffer D (pH 8.0, 10 mM Tris-HCl, 10 mM DTNB) was subsequently added. The mixture was incubated at 40 °C for 20 min and the absorbance was then determined at 412 nm. The content of sulfhydryl group was expressed as µmol/g protein.

### 2.2 Secondary structures

Fourier infrared spectrometer was used to determine the secondary structures of MP (Nicolet IS50, Thermo Scientific Inc., Waltham, MA, USA). After being freeze-dried, the samples were ground with potassium bromide powder for the following determination. The Gaussian curve fitting in Origin 2018 (OriginLab, Northampton, MA, USA) was used to deconvolute the spectra in the range from 1600 and 1700 cm^−1^, which represent the amide I region. The varied types of secondary structures were detected by dividing the peak area by the total area of the amide I area after deconvolution.

### 2.3 Tertiary structures

A fluorescence spectrophotometer in the emission scan mode was used to detect the tertiary structures of MP (F-7100, Hitachi, Tokyo, Japan). The imaging parameters were set as follows: emission wavelength was 305–410 nm, the excitation wavelength was 295 nm, scan speed was 1200 nm/min and slit width was 5 nm.

### 2.4 Confocal laser scanning microscopy (CLSM)

A Confocal laser scanning microscopy was used to obtain the MP aggregation images (Leica, SP8 SR, Germany). Referring to the approach of Tan, Ye, and Xie (2021b), the MP was physically labeled with a dimethyl sulfoxide (DMSO) solution of fluorochrome rhodamine B isothiocyanate (5 ppm). 10 μL of the samples were placed onto a slide to observe and the fluorescence intensity was recorded from 643 to 727 nm. The MP microstructure was imaged using a 10x water immersion objective lens. And all the captured digital images (1024 × 1024 pixel resolution) were saved in Tiff format.

### 2.5 Statistical analysis

Multiple comparisons were performed by one-way analysis of variance (ANOVA) followed by Duncan's test using SPSS 23.0. The mean ± standard deviation was used to report the experimental results.

## 3 Results and discussions

### 3.1 Freezing curve

**Fig. 2:**
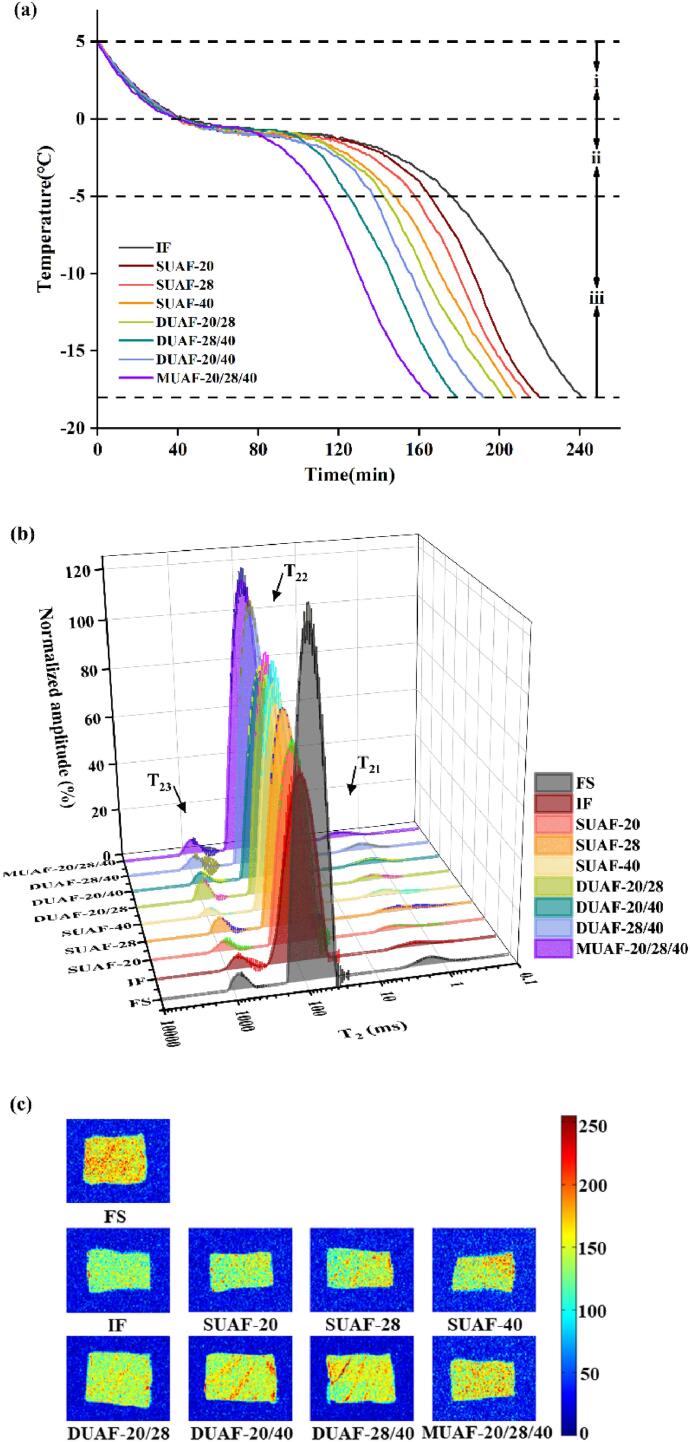
Freezing curves (a), water distribution (b) and magnetic resonance imaging (MRI) (c) of large yellow croaker with different freezing treatments, immersion freezing (IF), single-frequency UAF (SUAF) at 20 kHz, 28 kHz and 40 kHz, dual-frequency UAF (DUAF) at 20/28 kHz, 20/40 kHz and 28/40 kHz, multi-frequency UAF (MUAF) at 20/28/40 kHz. (For interpretation of the references to color in this figure legend, the reader is referred to the web version of this article.)

The temperature–time curve directly describes the freezing process and temperature changes of frozen samples under different freezing methods (Ma et al., 2021). The freezing rate is related to the distribution and size of ice crystals in frozen samples closely. The ice crystals produced by the rapid freezing method are smaller and more evenly distributed, to enhance the quality of frozen samples (Zhang et al., 2020b). The freezing process went through three stages: i) pre-freezing (5 to 0 °C), ii) phase change (0 to −5 °C) and iii) sub-freezing (−5 to −18 °C) stages (Zhu, Zhang, & Sun, 2020). The freezing time of IF was 241 min (Fig. 2a), while the freezing time of all the UAF treated samples was less than that of IF. The minimum reduction was 21 min (SUAF-20) and the maximum reduction was nearly 75 min (MUAF-20/28/40). UAF reduced the freezing time as the cavitation bubbles could act as the main ice cores. The mechanical force produced by the eruption of cavitation bubbles could break the original ice crystals into smaller sizes acting as the primary ice core (Qiu et al., 2022). The formation of plenty of small ice nuclei not only accelerated the growth of ice crystals and reduced the freezing time, but also made the formed ice crystals small and uniform, reducing the damage to muscles. Shi et al. (2019) also found UAF significantly accelerated the freezing process. Furthermore, multi-frequency UAF had higher freezing rates (Ye, Zhu, & Liu, 2019). Compared with single-frequency ultrasonic cavitation, the multi-frequency system promoted the cavitation nucleation and had the higher growth rate of the bubble and the collapse pressure (Ye, Zhu, He, & Song, 2021).

### 3.2 Thawing loss

Thawing loss is closely related to the water migration and the muscle tissue state of food (Table 1). And it is a vital indicator in analyzing the quality of frozen food (Li et al., 2017). The liquid lost after thawing is the result of the ice crystals damaging muscles by damaging cells and tissues during the freezing process (Stormo & Skåra, 2021). The thawing loss of MUAF was lower than IF, SUAF at 20 kHz, 28 kHz, 40 kHz, and DUAF at 20/28 kHz, 20/40 kHz, 28/40 kHz by 49.08%, 30.25%, 24.89%, 17.41%, 20.19%, 15.74%, and 7.26%, respectively. The difference was consistent with the trend of the freezing curve. MUAF had the lowest value of thawing loss due to the most regular and smallest ice crystals formed in the frozen samples. During the slow freezing process, large ice crystals formed could destroy fish tissue (Stormo & Skåra, 2021). The irregular distribution of these large ice crystals also led to the increase of extracellular space, the contraction of the myofilament lattice, and the denaturation of protein, resulting in high thawing loss (Dang et al., 2018). The thawed exudate contained a large number of water-soluble taste substances, such as amino acids or nucleotides, which led to the reduction of taste substances in thawed fish (Cartagena, Puértolas, & Martínez de Marañón, 2020). The increase in thawing loss also affected the flavor of fish and the lower thawing loss was favorable for the maintenance of fish flavor substances.

**Table 1:** The thawing loss and color results of frozen large yellow croaker under different methods.

Treatment	Thawing loss	Color			
	(%)	L*	a*	b*	ΔE
FS	–	41.44 ± 0.31^d^	2.47 ± 0.05^a^	3.60 ± 0.04^abc^	–
IF	3.26 ± 0.09^a^	49.51 ± 3.28^a^	2.50 ± 0.07^a^	−3.24 ± 0.04^a^	8.08 ± 2.97^a^
SUAF-20	2.38 ± 0.07^b^	46.79 ± 1.06^b^	2.53 ± 0.07^a^	3.49 ± 0.07^abc^	5.36 ± 0.82^b^
SUAF-28	2.21 ± 0.26^b^	45.70 ± 1.10^bc^	2.52 ± 0.03^a^	−3.30 ± 0.29^ab^	4.28 ± 0.88^bc^
SUAF-40	2.01 ± 0.01^b^	45.40 ± 0.70^bc^	2.54 ± 0.02^a^	3.38 ± 0.42^abc^	3.98 ± 0.52^bc^
DUAF-20/28	2.08 ± 0.03^b^	44.95 ± 0.60^bc^	2.49 ± 0.05^a^	−3.65 ± 0.12^bc^	3.51 ± 0.39^bc^
DUAF-20/40	1.97 ± 0.41^b^	44.60 ± 1.62^bc^	2.51 ± 0.06^a^	−3.76 ± 0.09 ^cd^	3.17 ± 1.32^bc^
DUAF-28/40	1.79 ± 0.18^b^	44.19 ± 0.75^bc^	2.48 ± 0.03^a^	−3.64 ± 0.18^bc^	2.76 ± 0.54^c^
MUAF-20/28/40	1.66 ± 0.26^b^	43.54 ± 1.33 ^cd^	2.53 ± 0.07^a^	3.51 ± 0.22^abc^	2.13 ± 1.01^c^

The methods include immersion freezing (IF), single-frequency UAF (SUAF) at 20 kHz, 28 kHz and 40 kHz, dual-frequency UAF (DUAF) at 20/28 kHz, 20/40 kHz and 28/40 kHz, multi-frequency UAF (MUAF) at 20/28/40 kHz. The letter from “a” to “d” are used to describe the significance of differences between the samples (p < 0.05).

### 3.3 Water distribution and migration

The LF-NMR transverse relaxation method is used to detect the water distribution and migration of samples (Fig. 2b). The relaxation time reflects the condition of hydrogen protons in large yellow croaker samples. And it is associated with the binding force and the degree of freedom of hydrogen protons, directly reflecting the distribution of water molecules (Bian, Cheng, Yu, Mei, & Xie, 2022). Therefore, it can directly reflect the water molecule distribution. The bound water, immobilized water, and free water were represented by the T_21_, T_22_, and T_23_ peaks, respectively (Sun, Zhang, Li, Xia, & Kong, 2020). There were no significant changes in the bound water content of fresh and thawed samples as shown in T_21_ areas indicating the close combination of bound water and protein (Tan, Ye, Chu, & Xie, 2021a). Immobilized water is the main form of water in the muscle of large yellow croakers, so the difference in T_22_ areas is the main basis for judging the water distribution and migration under different freezing treatment samples (Wang et al., 2021a). FS had the highest content of immobilized water and the frozen samples all had the lower content of immobilized water. Because of the destruction of the cell membrane by freeze–thaw, the damaged MP was unable to absorb extracellular dissolved water. This resulted in the conversion of partially immobilized water into free water (Wang et al., 2021a). IF had the lowest content of immobilized water, indicating that it has more serious damage to the MP structure. In addition, MUAF showed the highest immobilized water content, which was the closest to FS. It was consistent with the result of thawing loss that MUAF could better maintain the tissue structure of large yellow croakers and reduce water migration and loss.

MRI can intuitively reflect the changes in water migration of samples after different freezing methods (Fig. 2c). More obvious red indicates more water in this area, and the more obvious blue indicates less water (Sun, Zhang, Bhandari, & Yang, 2019). The images of all frozen samples were a little bluer and darker than FS, which demonstrated that the water in frozen samples had migrated and lost. Tan et al. (2021a) also found the MRI images of the frozen large yellow croaker turned less red and bluer. Moreover, the difference between MUAF and FS was minimal, this was consistent with LF-NMR results. It might be due to the smaller ice crystals generated under MUAF treatment, and it caused less damage to muscle fibers and MP structure.

### 3.4 Color

The color of samples is an intuitive indicator of consumers' choice and an important factor in determining the freshness of fish (Duan et al., 2020). All the frozen samples had an increase in the value of L* compared to FS (Table 1). The ice crystals developed in the freezing process and liquid loss caused by the ice crystal melting during the thawing process were the main reasons for the changes in L* values (Bian et al., 2022). During the freezing process, the ice crystals were formed in the gaps between the muscle tissue, and they melted as the samples thawed. Light scattering caused by ice crystals also was responsible for the changes in L* values (Cai, Wan, Li, & Li, 2020). IF had the highest L* value, which was due to the highest content of surface free water caused by the liquid loss (Qiu, Zhang, Chitrakar, & Bhandari, 2020). In addition, the L* value of MUAF was closest to that of FS, which might be since rapid freezing protects the muscle tissue of the samples and reduces the liquid loss, leading to less water content on the sample surface and a lower L* value. In addition, there was no significant difference between the differently treated samples about a* and b* values. Ma et al. (2021) concluded that freezing did not influence on a* and b* values when studying the quality of frozen large yellow croakers. ΔE represents the magnitude of the total chromatic aberration, perceptible to the naked eye on a scale from 2 to 10 (Bian et al., 2022). In this study, the ΔE values of the IF and SUAF-20 group were greater than 5, indicating that the color changes of the samples could be detected by the naked eyes. In addition, MUAF had the lowest ΔE value at 2.13 and the color in the MUAF treated samples was close to FS.

### 3.5 TVB-N and TBARS

TVB-N is the main indicator to judge the freshness of fish, which digitally represents the volatile basic compound content (Jiang et al., 2020) (Table 2). It includes trimethylamine, ammonia, dimethylamine, methylamine, etc., which are generated by the degradation of non-protein nitrogen compounds and protein (Sun, Chen, Xia, Kong, & Diao, 2019b). The TVB-N value of FS was 9.81 ± 0.03 mg N/100 g. All the frozen samples had an increased value of TVB-N. During the freezing process, spoilage induced by enzymes led to the degradation of nitrogen-containing compounds and proteins. That led to the accumulation of the organic amine, as shown by increasing the TVB-N value (Bian et al., 2022). The highest TVB-N value appeared on IF, indicating the poor freshness. MUAF obtained the lowest TVB-N value in the frozen samples and was closest to the freshness state of FS.

**Table 2:** The total volatile basic nitrogen (TVB-N), thiobarbituric acid reactive substances (TBARS), carbonyl content, and total sulfhydryl content results of frozen large yellow croaker under different methods.

Treatment	TVB-N	TBARS	Carbonyl content	Total sulfhydryl content
	(mg N/100 g)	(10^−2^ mg/MDA kg)	(μmol/g protein)	(μmol/g protein)
FS	9.81 ± 0.03^f^	11.67 ± 0.06 ^g^	2.22 ± 0.07 ^g^	72.02 ± 0.44^a^
IF	10.81 ± 0.08^a^	14.70 ± 0.30^a^	3.01 ± 0.02^a^	56.19 ± 2.58^f^
SUAF-20	10.62 ± 0.04^b^	13.63 ± 0.35^b^	2.91 ± 0.05^b^	59.72 ± 1.05^e^
SUAF-28	10.51 ± 0.05^c^	13.30 ± 0.10^c^	2.88 ± 0.05^b^	60.62 ± 1.36^e^
SUAF-40	10.47 ± 0.06^c^	13.10 ± 0.17 ^cd^	2.83 ± 0.04^bc^	61.64 ± 0.49^de^
DUAF-20/28	10.34 ± 0.03^d^	12.83 ± 0.12^de^	2.76 ± 0.06 ^cd^	63.15 ± 0.95 ^cd^
DUAF-20/40	10.32 ± 0.03^d^	12.73 ± 0.06^e^	2.72 ± 0.03^de^	64.03 ± 0.28^c^
DUAF-28/40	10.28 ± 0.03^d^	12.37 ± 0.06^f^	2.67 ± 0.04^e^	64.92 ± 0.20^c^
MUAF-20/28/40	10.09 ± 0.05^e^	12.07 ± 0.15^f^	2.42 ± 0.06^f^	67.13 ± 1.04^b^

The methods include immersion freezing (IF), single-frequency UAF (SUAF) at 20 kHz, 28 kHz and 40 kHz, dual-frequency UAF (DUAF) at 20/28 kHz, 20/40 kHz and 28/40 kHz, multi-frequency UAF (MUAF) at 20/28/40 kHz. The letter from “a” to “g” are used to describe the significance of differences between the samples (p < 0.05).

The formation of oxygen free radicals and/or lipid-free radicals caused lipid oxidation, which led to the production of toxic compounds such as cholesterol and malondialdehyde (Abdel-Naeem, Sallam, & Malak, 2021). The TBARS value of FS was 11.67 ± 0.06 × 10^−2^ mg/MDA kg (Table 2). Although freezing could put off the progress of various biochemical reactions in fish to a certain degree, some oxidative degradations still existed (Cheng, Sørensen, Engelsen, Sun, & Pu, 2019). Therefore, all the UAF samples significantly had higher TBARS values than FS (p < 0.05), which was owing to the formation of the ice crystals during freezing. The ice crystals damaged the muscle cells and facilitated the release of oxidation precursors to promote the oxidation reaction (Li et al., 2020). The TBARS value of the UAF samples was significantly lower than that of IF significantly. This indicated UAF could effectively reduce the lipid oxidation of frozen large yellow croakers. The reasons for the lower lipid oxidation in UAF might be attributed to the following two points: i) the small-size and evenly distributed ice crystals developed in the rapid freezing had a smaller contact area with oxygen after thawing; ii) UAF could restrain the lipid oxidation by controlling the activities of phospholipase, lipase, and lipoxygenase (Sun et al., 2019b). MUAF had the lowest TBARS value at 12.07 ± 0.15 × 10^−2^ mg/MDA kg. MUAF had a faster freezing rate, resulting in that the samples maintaining quality characteristics better. In addition, the higher ultrasonic intensity in MUAF also inhibited the activity of enzymes.

### 3.6 Carbonyl content

The oxidation of protein can result in the breaking of the main chain, the development of cross-linking, and the transformation of some amino acid residues into carbonyl derivatives (Sun et al., 2019b). During fish processing, amino acid residues containing free amines (ε-NH_2_ samples) such as proline, lysine, threonine, and arginine can be converted to carbonyl samples through the process of deamination (Li, Li, Hu, Chen, & Li, 2014). Therefore, the carbonyl content of MP is an indicator to judge the severity of protein oxidation (Table 2). Compared with FS, the carbonyl content of all the frozen samples aggrandized significantly (p < 0.05). The increase of total free carbonyl content indicated that MP was oxidized, which caused oxidative degradation of histidine, proline, arginine, and lysine residues (Qiu et al., 2022). Amino acids such as lysine, proline, and arginine are easily oxidized into semialdehyde under the action of reactive oxygen species and metal ions, accounting for most of the total carbonyl contents (Li et al., 2021). Hu et al. (2021) discovered that the carbonyl content of hairtail MP had an increase after liquid nitrogen quick-freezing, confirming the oxidation of frozen fish protein. The carbonyl content of IF was higher than that of UAF samples significantly. It indicated that MP in IF was more prone to oxidation, which was unfavorable to the stability of fish protein (Guyon, Meynier, & de Lamballerie, 2016). Protein denaturation also negatively affects the solubility, gel properties and water retention of proteins (Wu, Zhang, Jia, Kuang, & Yang, 2018). In contrast, MUAF showed significant advantages in maintaining protein stability and the carbonyl content of MUAF was closest to that of FS. The trends were similar to that of TBARS values, which due to the reactions of the protein oxidation and the lipid oxidation were very complex and difficult to distinguish from each other (Yang et al., 2019).

### 3.7 Total sulfhydryl content

Sulfhydryl is generally considered to be a significant indicator in evaluating the extent of protein denaturation and unfolding because it is sensitive to active hydroxyl samples (Oliveira, Neto, Santos, Ferreira, & Rosenthal, 2017) (Table 2). The total sulfhydryl content decrease with the increase of protein oxidation (Li et al., 2021). The sulfhydryl content of samples under UAF treatments decreased irreversibly compared with FS. The freezing of the free water caused a reduction in the water activity and a relative increase of the solute in unfrozen water, resulting in more effective contact between reactants. MP is attached to plenty of unfrozen water, and an oxidant is enriched in water, indicating that freezing accelerates the oxidation of the oxidant (Sun, Kong, Liu, Zheng, & Zhang, 2021). Zheng, Zhou, Zhang, Wang, and Wang (2021) also found that protein oxidation occurred in obscure pufferfish (*Takifugu obscurus*) in the frozen storage, and the content of the sulfhydryl group decreased significantly. The sulfhydryl samples could be oxidized and transformed into the disulfide samples, resulting in a reduction of sulfhydryl content. Disulfide bonds produced by sulfhydryl oxidation could not only cause the aggregation between proteins through cross-linking, but also had a serious influence on the process of freezing-induced protein denaturation (Benjakul, Visessanguan, Thongkaew, & Tanaka, 2003). Besides, the sulfhydryl sample masking caused by protein aggregation also resulted in a reduction in sulfhydryl content (Gao, Huang, Zeng, & Brennan, 2019). Among the frozen samples, the sulfhydryl content of IF (56.19 ± 2.58 μmol/g protein) decreased, while the oxidation degree of MUAF was the lowest. The sulfhydryl content of MUAF was 67.13 ± 1.04 μmol/g protein, which was 12.41%, 10.74%, 8.91%, 6.30%, 4.84%, and 3.40% higher than that of SUAF at 20 kHz, 28 kHz, 40 kHz, and DUAF at 20/28 kHz, 20/40 kHz, 28/40 kHz, respectively. MUAF effectively inhibited the loss of sulfhydryl content as it maximized the completeness of fish muscle cells and decreased the oxidase releasing in cells to reduce the degree of oxidation reactions (Sun et al., 2021).

### 3.8 Secondary structures

**Fig. 3:**
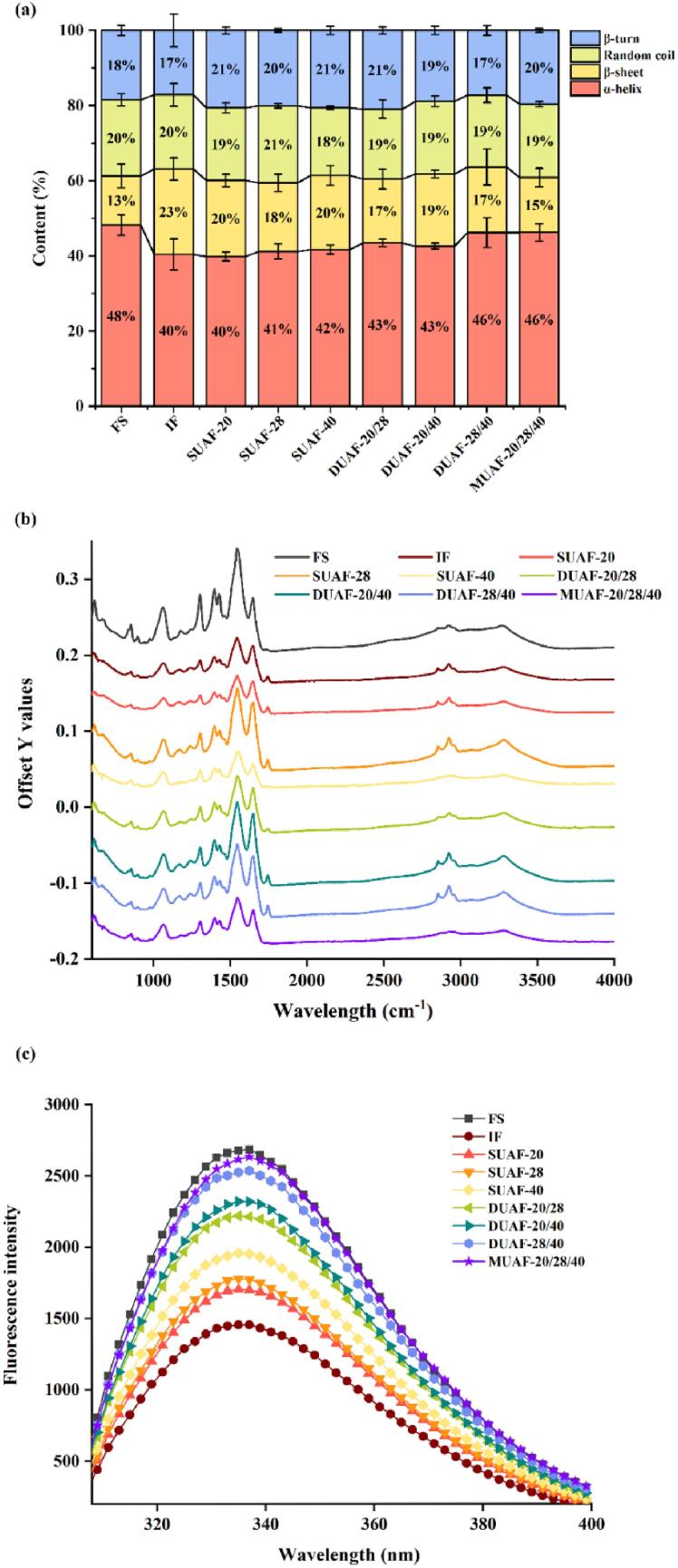
The difference of secondary structure (a) (b) and tertiary structure (c) of protein of large yellow croaker with different freezing treatments, immersion freezing (IF), single-frequency UAF (SUAF) at 20 kHz, 28 kHz and 40 kHz, dual-frequency UAF (DUAF) at 20/28 kHz, 20/40 kHz and 28/40 kHz, multi-frequency UAF (MUAF) at 20/28/40 kHz. (For interpretation of the references to color in this figure legend, the reader is referred to the web version of this article.)

The influence of different freezing methods on the secondary structures of the protein was studied by the FT-IR spectroscopy (Xu et al., 2020) (Fig. 3a and b). The protein mass spectrum included the amide I region, the amide II region, and the water region. The amide I band could be used as an indicator of changes in protein secondary structures because of its high sensitivity. It was mainly based on the symmetrical stretching vibration of carbonyl (C=O) functional samples, including α-helix, β-sheet, random coil, and β-turn (Zhang, Li, Wang, Xia, & Kong, 2020a). The changes of α-helix, β-sheet, random coil, and β-turn content were shown in Fig. 3(a). The contents of α-helix in all the frozen samples decreased and the contents of β-sheet increased compared with FS. The change of IF group was the largest, reflecting that fish protein under IF treatment was severely damaged. The α-helix was the main secondary structure, and the content of it was reduced contributing to the unfolding of the protein (Zhang et al., 2020). Therefore, the content changes of frozen samples were directly proportional to the degree of protein oxidation. The severe protein oxidation led to the degradation in the secondary structures. Furthermore, the secondary structure changes of UAF samples were also due to UAF altering the spatial entanglement of protein molecules. It caused the structural loss, dissolved part of the protein, exposed the protein residue to water molecules, formed new hydrogen bonds, and made the α-helix structure turn into a β-sheet structure (Wang, Tayyab Rashid, Yan, & Ma, 2021b). The decrease of α-helix content will also lead to more exposure of hydrophobic groups and increase of surface hydrophobicity, resulting in higher drip loss (Zhao et al., 2019). Sun, Chen, Xia, Kong, and Diao (2019a) discovered that UAF with appropriate parameters could reduce the changes of MP secondary structures. Since the secondary structures of MUAF was closest to FS, it could be assumed that MUAF could greatly keep the structural characteristics of large yellow croaker protein and make the fish protein more stable. Since the secondary structures of MUAF was closest to that of FS, it could be assumed that MUAF better maintained the structure of large yellow croaker protein and made the fish protein more stable.

### 3.9 Tertiary structures

Endogenous fluorescence spectroscopy is widely used to measure tryptophan residues with the aim of evaluating the changes in protein tertiary structures (Du et al., 2021). Aromatic amino acid residues are the main contributors to the intrinsic fluorescence of proteins, such as tryptophan, tyrosine, and phenylalanine. These aromatic amino acid residues produce fluorescence at a specific excitation wavelength, and the position of aromatic amino acid residues (especially tryptophan) in proteins affects the fluorescence energy (Tan et al., 2022). The fluorescence intensity of FS was the highest (Fig. 3c). Freezing resulted in partial exposure of the tyrosine residue to the protein surface, leading to some variations in the tertiary structures of MP. Therefore, the fluorescence intensities of all the UAF samples had reduced (Zhang et al., 2020a). During the frozen samples, the fluorescence intensity of IF was the lowest as the slow freezing resulted in larger ice crystals to cause greater damage to the muscle tissue and accelerate the oxidation and destruction of MP structures. Sun et al. (2019a) also demonstrated that the UAF treatment reduced the damage to frozen common carp and resulted in better MP tertiary structures.

### 3.10 CLSM

**Fig. 4:**
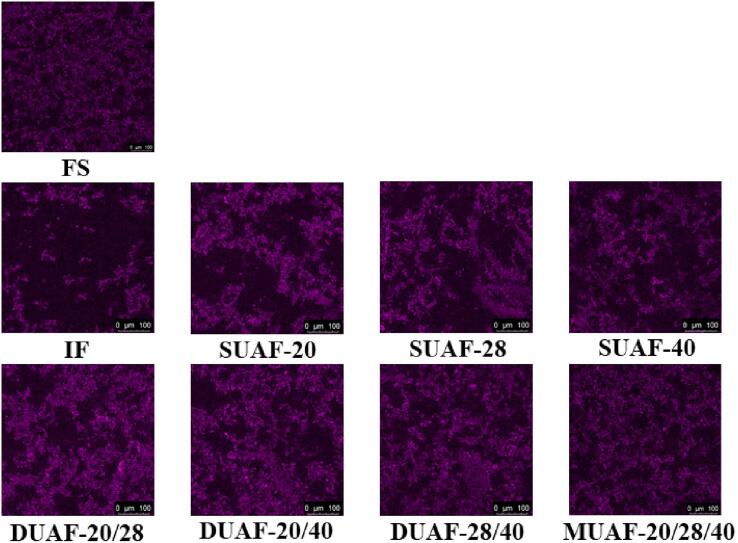
Confocal laser scanning microscopy (CLSM) images of large yellow croaker with different freezing treatments, immersion freezing (IF), single-frequency UAF (SUAF) at 20 kHz, 28 kHz and 40 kHz, dual-frequency UAF (DUAF) at 20/28 kHz, 20/40 kHz and 28/40 kHz, multi-frequency UAF (MUAF) at 20/28/40 kHz. (For interpretation of the references to color in this figure legend, the reader is referred to the web version of this article.)

The microstructures of MP suspension showed different changes under different freezing conditions (Fig. 4). In the image, the bright area stained with rhodamine isothiocyanate B showed the MP, while the dark area corresponded to the MP-free area. The MP distribution of FS was relatively uniform, while the MP of the frozen samples showed aggregation and uneven distribution. Freezing destroyed the conformation of MP and induced the formation of MP aggregation. Tan et al. (2021b) also found the aggregation phenomenon of frozen fish MP in the frozen large yellow croaker. Furthermore, the higher stability of the MP in UAF samples was due to the gradual transformation of the filamentous polymer in the MP solution turned into small units and had a more uniform distribution (Liu, Zhang, Wang, Chen, & Kong, 2021). MUAF had the closest MP state to FS, which had always shown the advantage of maintaining the protein structure stable in large yellow croakers.

### 3.11 Possible mechanisms in UAF

**Fig. 5:**
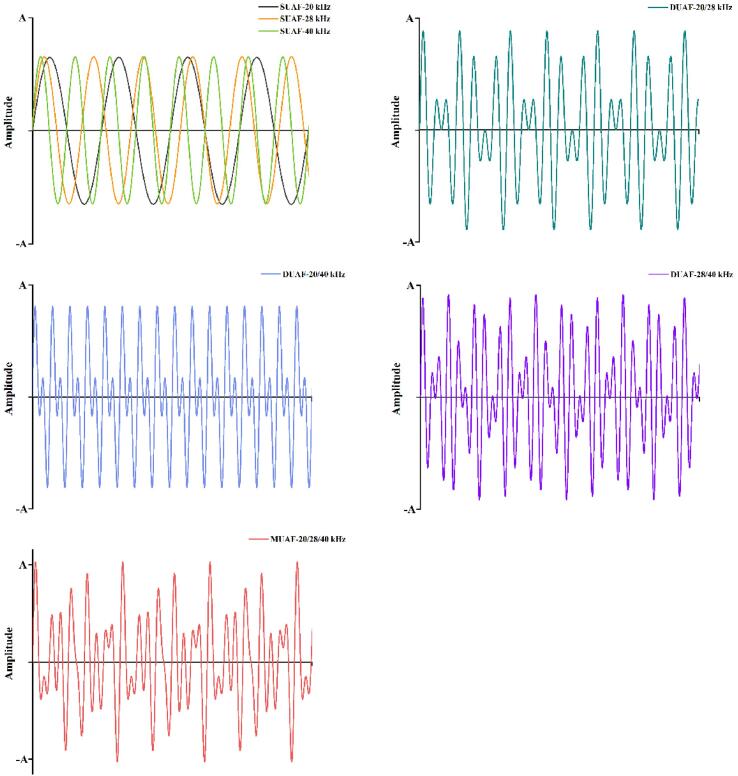
Possible mechanisms of ultrasound assisted freezing (UAF) groups.

The possible mechanisms in UAF groups were shown in Fig. 5. Ultrasound could facilitate the freezing process, mainly because it participates in the primary and secondary nucleation of ice crystals (Bhargava, Mor, Kumar, & Sharanagat, 2021). The primary nucleation was accompanied by the release of plenty of latent heat. The high pressure caused by ultrasound reduced the undercooling and promotes the nucleation of ice crystals (Ye et al., 2021). Ultrasonic cavitation bubbles could also act as ice nuclei during freezing to accelerate the development of ice crystals (Zhang et al., 2020b). The movement of stable cavitation bubbles could lead to microflow and eddy current, which enhanced the heat and mass transfer, resulting in nucleation (Cavalaro et al., 2019, Qiu et al., 2020). Due to the burst of cavitation bubbles and the shear force produced by microflow, the large ice crystals were decomposed into smaller ice crystals, accelerating the secondary nucleation process (Bhargava et al., 2021). The bubble-burst energy had a close relationship with the ultrasonic cavitation effect (Wang, Ning, Lv, Yao, & Sun, 2021). Dual-frequency and multi-frequency had synergistic effects, and the increased influence of bubble-burst energy in dual-frequency and multi-frequency UAF-driven cavitation bubbles was one of the potential mechanisms (Feng, Zhao, Zhu, & Mason, 2002).

## 4 Conclusions

The research evaluated the effects of UAF on the freezing speed, muscle quality, and MP structure of the large yellow croakers. UAF significantly promoted the freezing process of large yellow croakers, and MUAF had the fastest freezing rate. MUAF maintained better quality characteristics than other frozen samples and had lower thawing loss and water migration. The samples treated with MUAF had the closest color to FS. MUAF also had a lower TBARS and TVB-N value, which meant a lower degree of lipid oxidation and better freshness. In addition, MUAF had lower carbonyl content and higher sulfhydryl content, indicating a lower MP oxidation degree. It could also be found from the infrared detection and fluorescence intensity analysis that MUAF had better second structures and more stable tertiary structures. It could be concluded that MUAF effectively reduced the damage of freezing to MP, better maintained the stability of fish protein, and made its quality similar to that of FS. Therefore, the above results showed that MUAF was an efficient method to facilitate the freezing process and enhance the quality and the MP structure characteristics of large yellow croakers.

## Funding

This research was financially supported by China Agriculture Research System of MOF and MARA (CARS-47), and the Shanghai Professional Technology Service Platform on Cold Chain Equipment Performance and Energy Saving Evaluation (20DZ2292200, 19DZ1207503).

All authors have read and agreed to the published version of the manuscript.

### CRediT authorship contribution statement

**Chuhan Bian:** Conceptualization, Methodology, Software, Investigation. **Huijie Yu:** Methodology, Investigation. **Kun Yang:** Conceptualization, Software. **Jun Mei:** Validation, Formal analysis, Writing – review & editing. **Jing Xie:** Funding acquisition.

## Declaration of Competing Interest

The authors declare that they have no known competing financial interests or personal relationships that could have appeared to influence the work reported in this paper.
